# Durability of Gutta Percha Fillings

**Published:** 1882-02

**Authors:** 


### EDITORIAL, ETC.

*Durability of Gutta Percha Fillings.*—Although looked upon ordinarily as a filling of quite temporary character, yet the occasional endurance of gutta-percha has proved remarkable under some severe trials. A few fillings were renewed lately by the writer from cavities on the buccal walls of lower and upper molars, which fillings were in good condition and only removed because of the extension of the decay further around the tooth, twelve and fifteen years old. The integrity of the gutta-percha seemed perfect, and no special softening of the cavity under the filling was noticed. The writer has for some time been in the habit of making compound fillings of gutta-percha and oxychloride or oxyphosphate in proximal cavities by filling the cervical third of the cavity with gutta-percha and finishing with the zinc filling. Its use thus seems a good one, as the failure of the zinc filling next the gum is so frequent, and the gutta-percha at that part is well protected. Of late too, though not sufficiently long to prove much, we filled this part of the cavity with tin foil to a point on a line with the gum, finishing with gold. The tin shows slightly if at all, and the promise is of greater durability.

